# Supplementary material for: LIN28A-Dependent Kinome and Phosphoproteome Reprogramming Promotes Imatinib Resistance
Source: Mol Cell Proteomics. 2026 Jan 19;25(4):101514. doi: 10.1016/j.mcpro.2026.101514 (PMC13091335; doi:10.1016/j.mcpro.2026.101514)
Supplement: Supplemental data [file mmc4.pptx]

## Slide 1
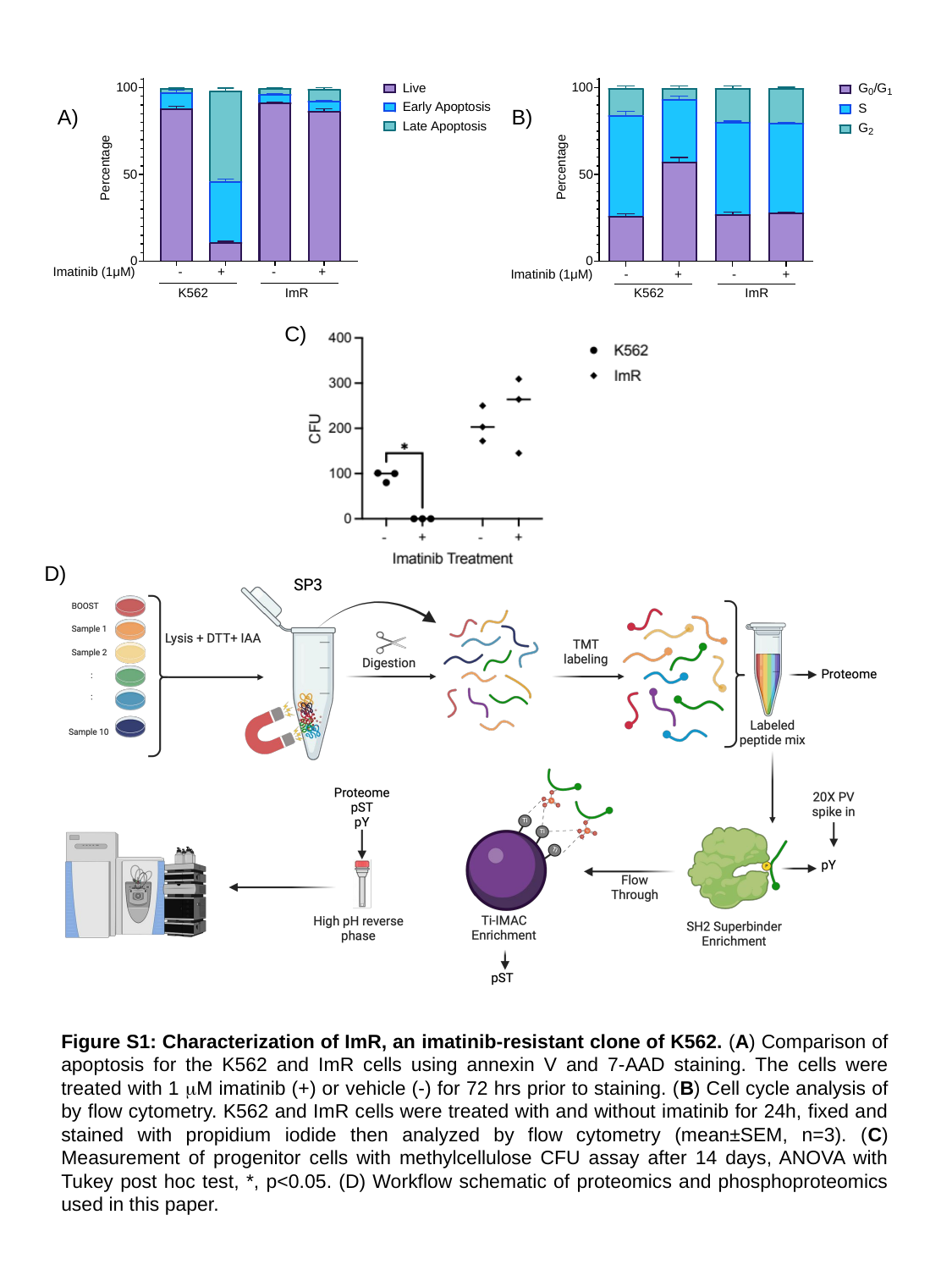

B)
A)
C)
D)
Figure S1: Characterization of ImR, an imatinib-resistant clone of K562. (A) Comparison of apoptosis for the K562 and ImR cells using annexin V and 7-AAD staining. The cells were treated with 1 mM imatinib (+) or vehicle (-) for 72 hrs prior to staining. (B) Cell cycle analysis of by flow cytometry. K562 and ImR cells were treated with and without imatinib for 24h, fixed and stained with propidium iodide then analyzed by flow cytometry (mean±SEM, n=3). (C) Measurement of progenitor cells with methylcellulose CFU assay after 14 days, ANOVA with Tukey post hoc test, *, p<0.05. (D) Workflow schematic of proteomics and phosphoproteomics used in this paper.

## Slide 2
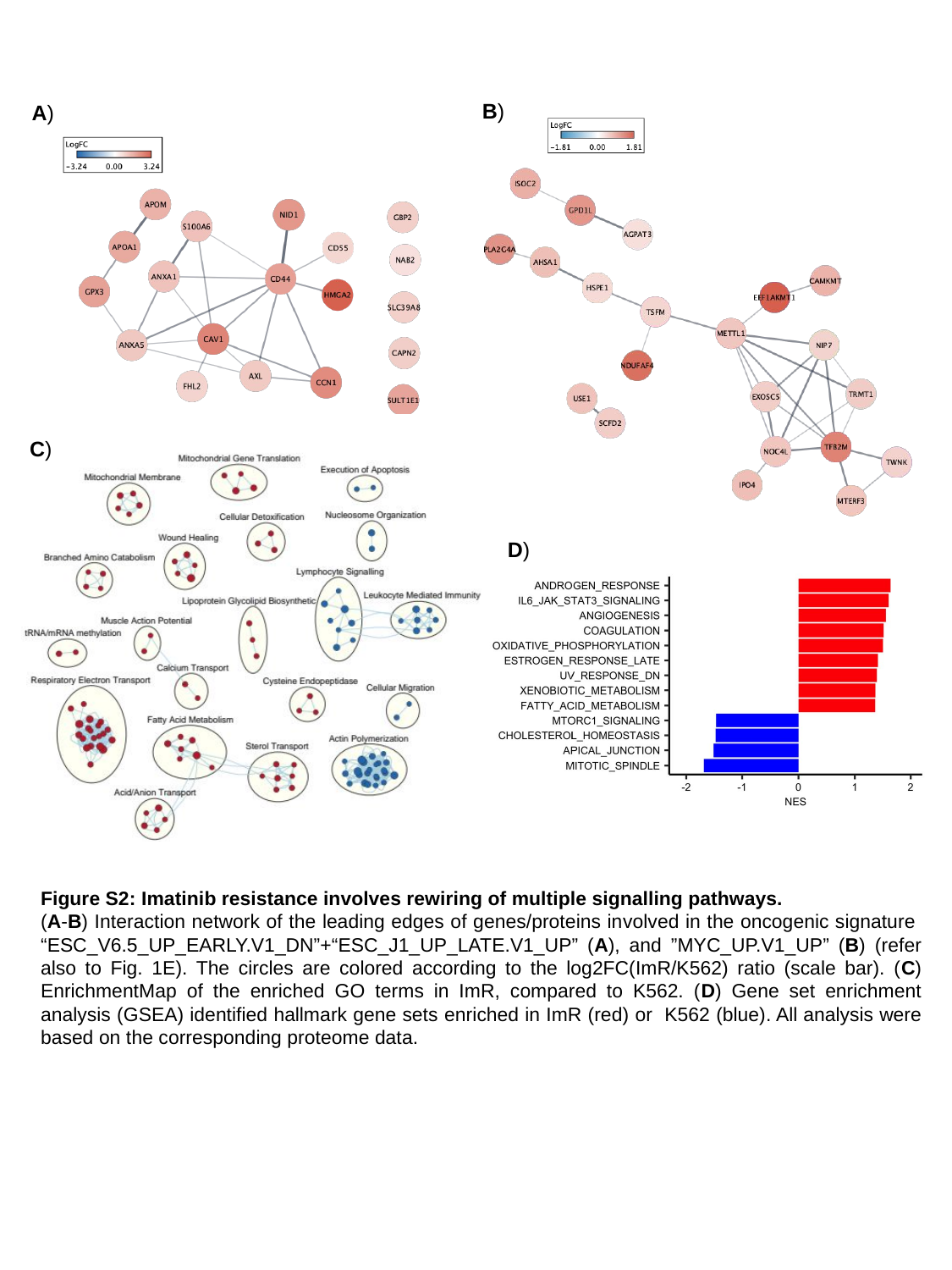

B)
A)
C)
D)
Figure S2: Imatinib resistance involves rewiring of multiple signalling pathways.
(A-B) Interaction network of the leading edges of genes/proteins involved in the oncogenic signature “ESC_V6.5_UP_EARLY.V1_DN”+“ESC_J1_UP_LATE.V1_UP” (A), and ”MYC_UP.V1_UP” (B) (refer also to Fig. 1E). The circles are colored according to the log2FC(ImR/K562) ratio (scale bar). (C) EnrichmentMap of the enriched GO terms in ImR, compared to K562. (D) Gene set enrichment analysis (GSEA) identified hallmark gene sets enriched in ImR (red) or K562 (blue). All analysis were based on the corresponding proteome data.

## Slide 3
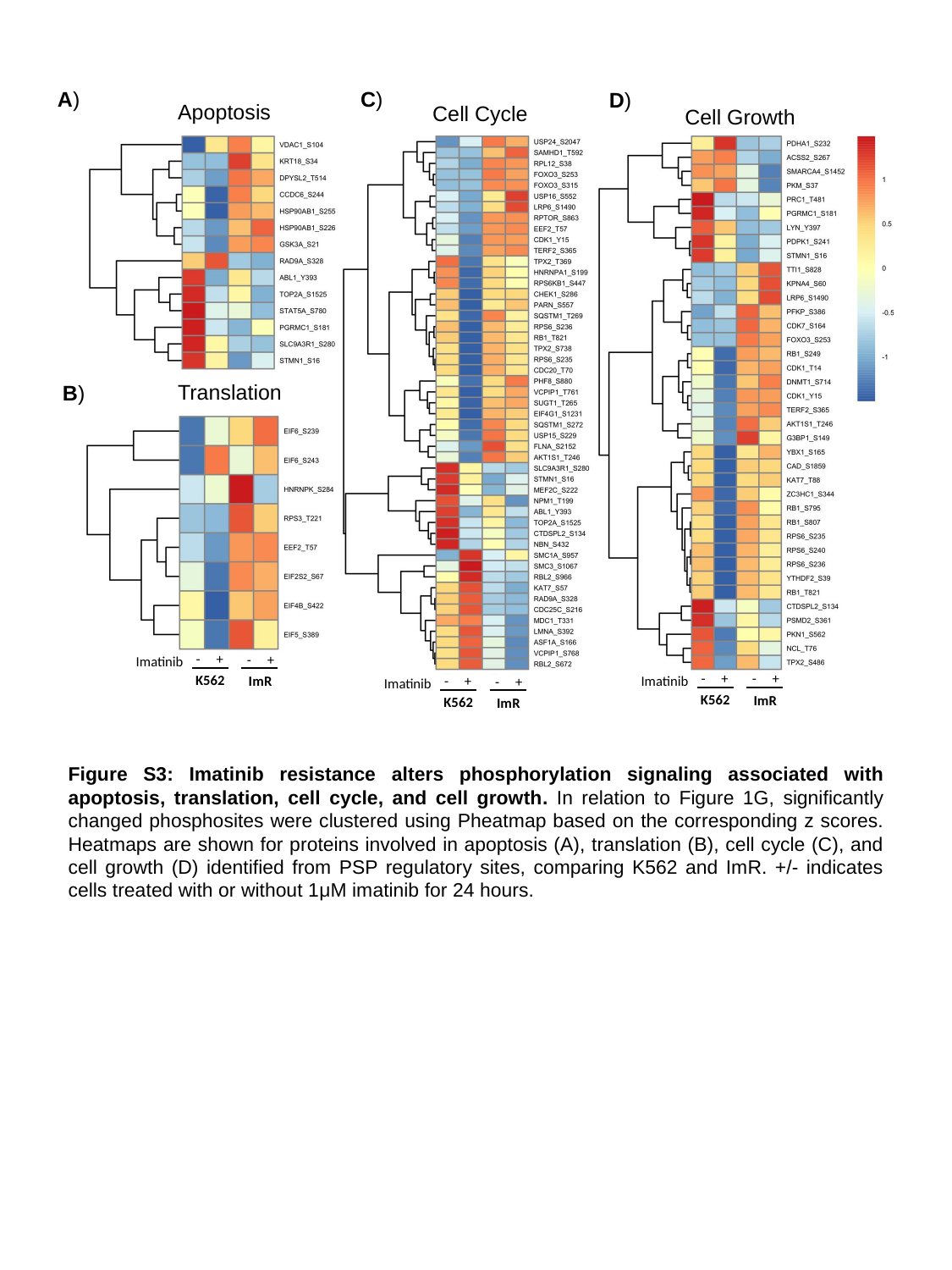

A)
C)
D)
Apoptosis
Cell Cycle
Cell Growth
Translation
B)
- +
- +
Imatinib
- +
- +
K562
Imatinib
- +
ImR
- +
Imatinib
K562
ImR
K562
ImR
Figure S3: Imatinib resistance alters phosphorylation signaling associated with apoptosis, translation, cell cycle, and cell growth. In relation to Figure 1G, significantly changed phosphosites were clustered using Pheatmap based on the corresponding z scores. Heatmaps are shown for proteins involved in apoptosis (A), translation (B), cell cycle (C), and cell growth (D) identified from PSP regulatory sites, comparing K562 and ImR. +/- indicates cells treated with or without 1μM imatinib for 24 hours.

## Slide 4
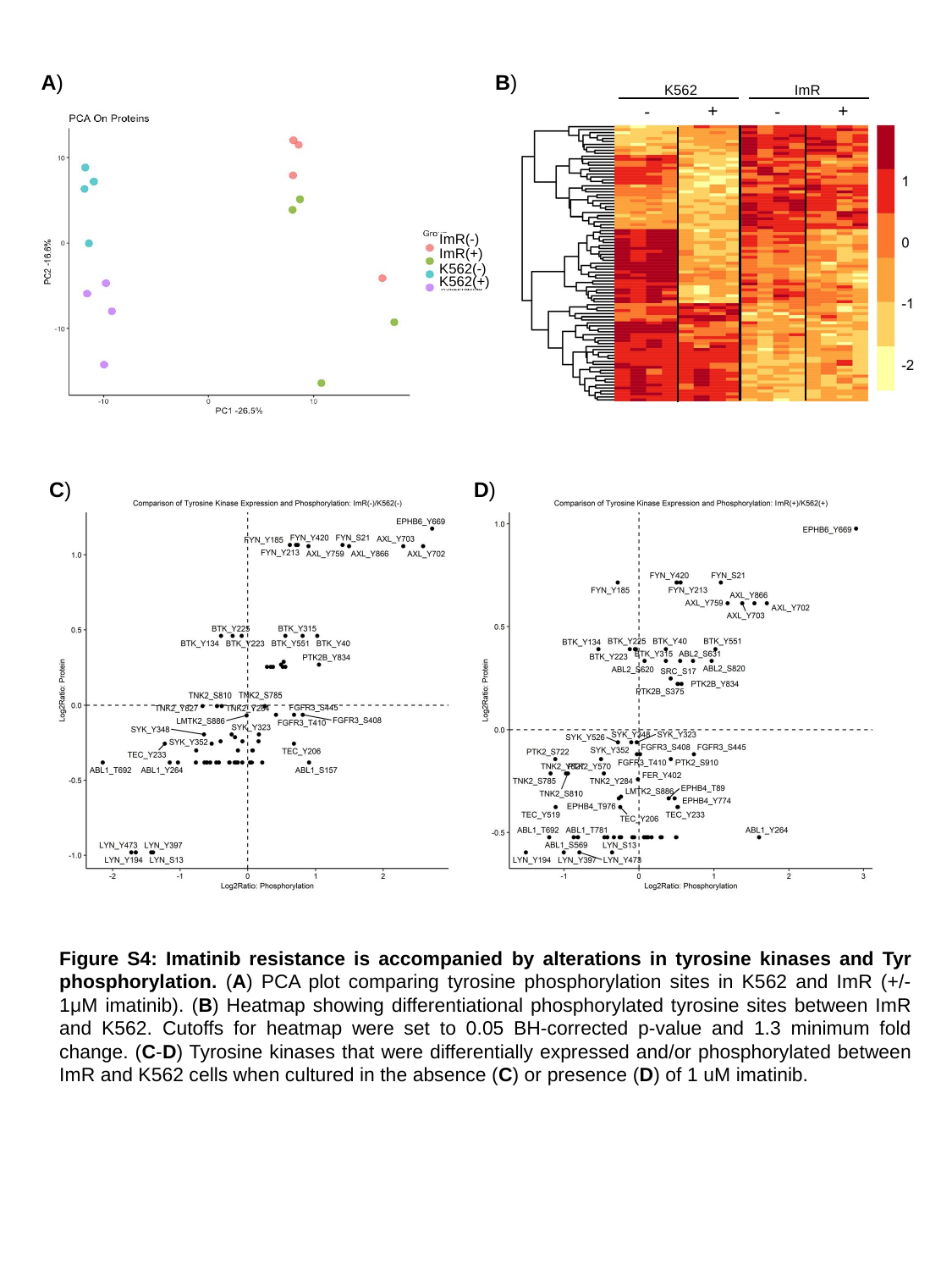

A)
B)
K562
+
-
ImR
+
-
ImR(-)
ImR(+)
K562(-)
K562(+)
C)
D)
Figure S4: Imatinib resistance is accompanied by alterations in tyrosine kinases and Tyr phosphorylation. (A) PCA plot comparing tyrosine phosphorylation sites in K562 and ImR (+/- 1μM imatinib). (B) Heatmap showing differentiational phosphorylated tyrosine sites between ImR and K562. Cutoffs for heatmap were set to 0.05 BH-corrected p-value and 1.3 minimum fold change. (C-D) Tyrosine kinases that were differentially expressed and/or phosphorylated between ImR and K562 cells when cultured in the absence (C) or presence (D) of 1 uM imatinib.

## Slide 5
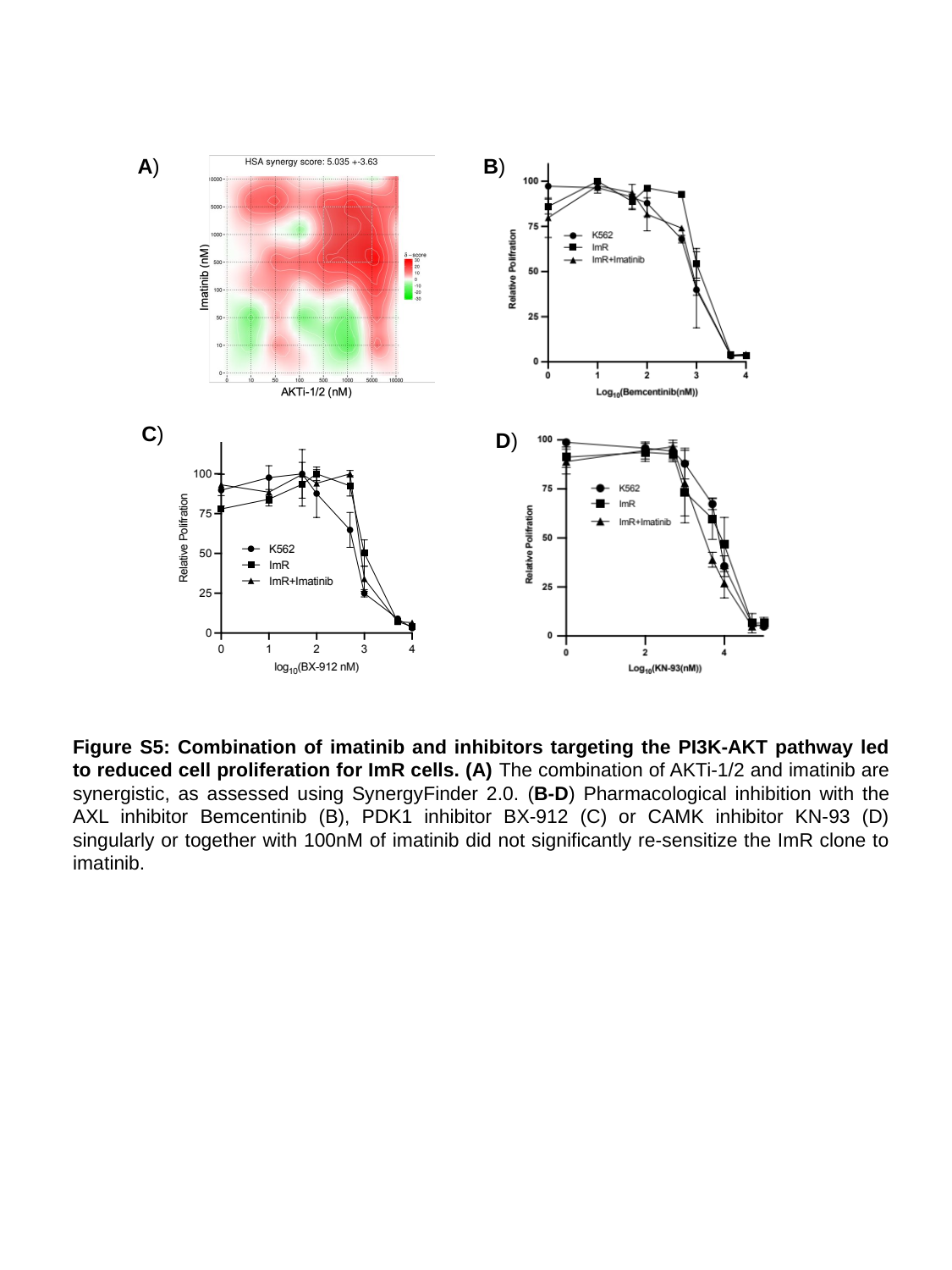

B)
A)
C)
D)
Figure S5: Combination of imatinib and inhibitors targeting the PI3K-AKT pathway led to reduced cell proliferation for ImR cells. (A) The combination of AKTi-1/2 and imatinib are synergistic, as assessed using SynergyFinder 2.0. (B-D) Pharmacological inhibition with the AXL inhibitor Bemcentinib (B), PDK1 inhibitor BX-912 (C) or CAMK inhibitor KN-93 (D) singularly or together with 100nM of imatinib did not significantly re-sensitize the ImR clone to imatinib.

## Slide 6
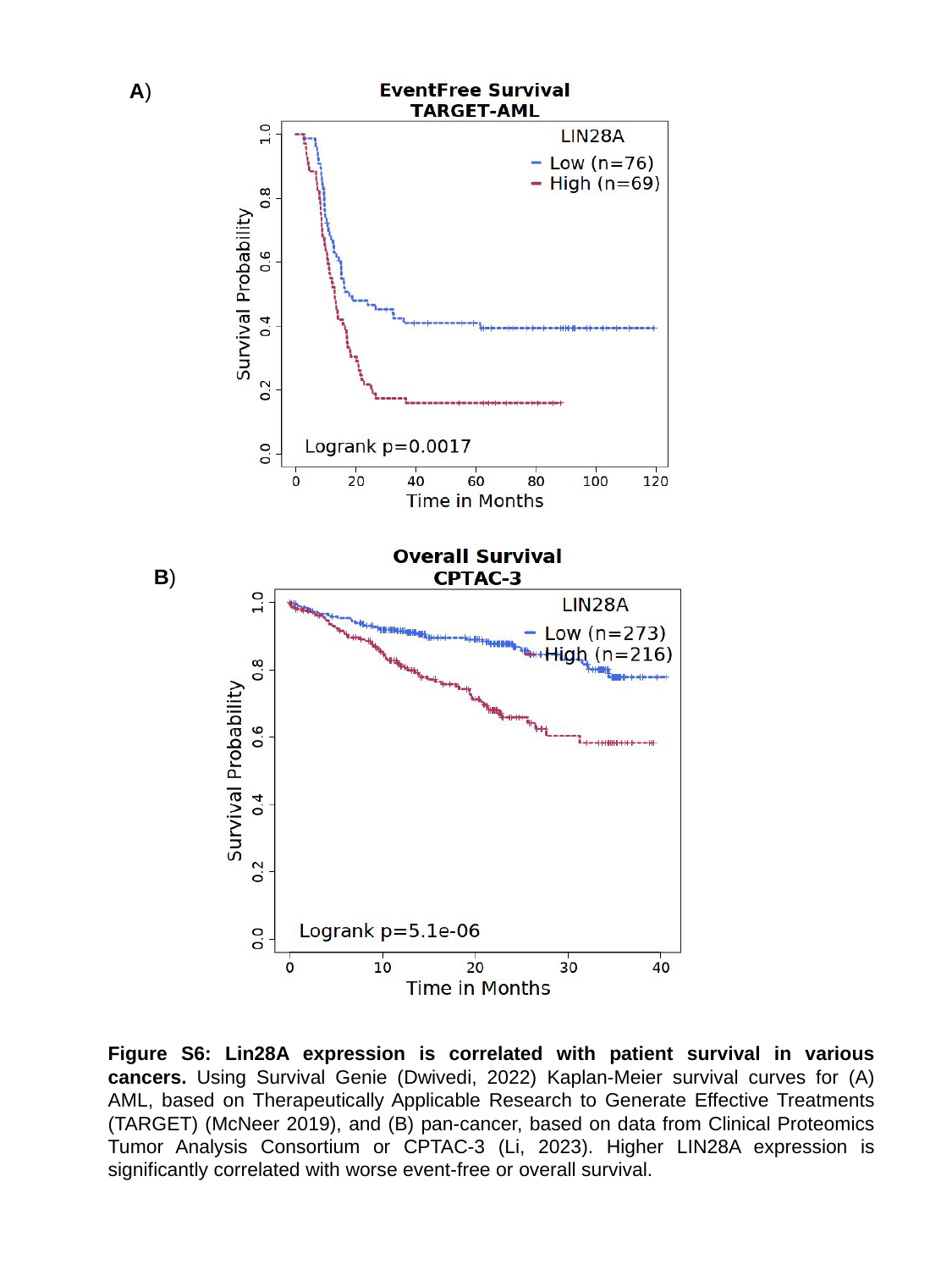

A)
B)
Figure S6: Lin28A expression is correlated with patient survival in various cancers. Using Survival Genie (Dwivedi, 2022) Kaplan-Meier survival curves for (A) AML, based on Therapeutically Applicable Research to Generate Effective Treatments (TARGET) (McNeer 2019), and (B) pan-cancer, based on data from Clinical Proteomics Tumor Analysis Consortium or CPTAC-3 (Li, 2023). Higher LIN28A expression is significantly correlated with worse event-free or overall survival.

## Slide 7
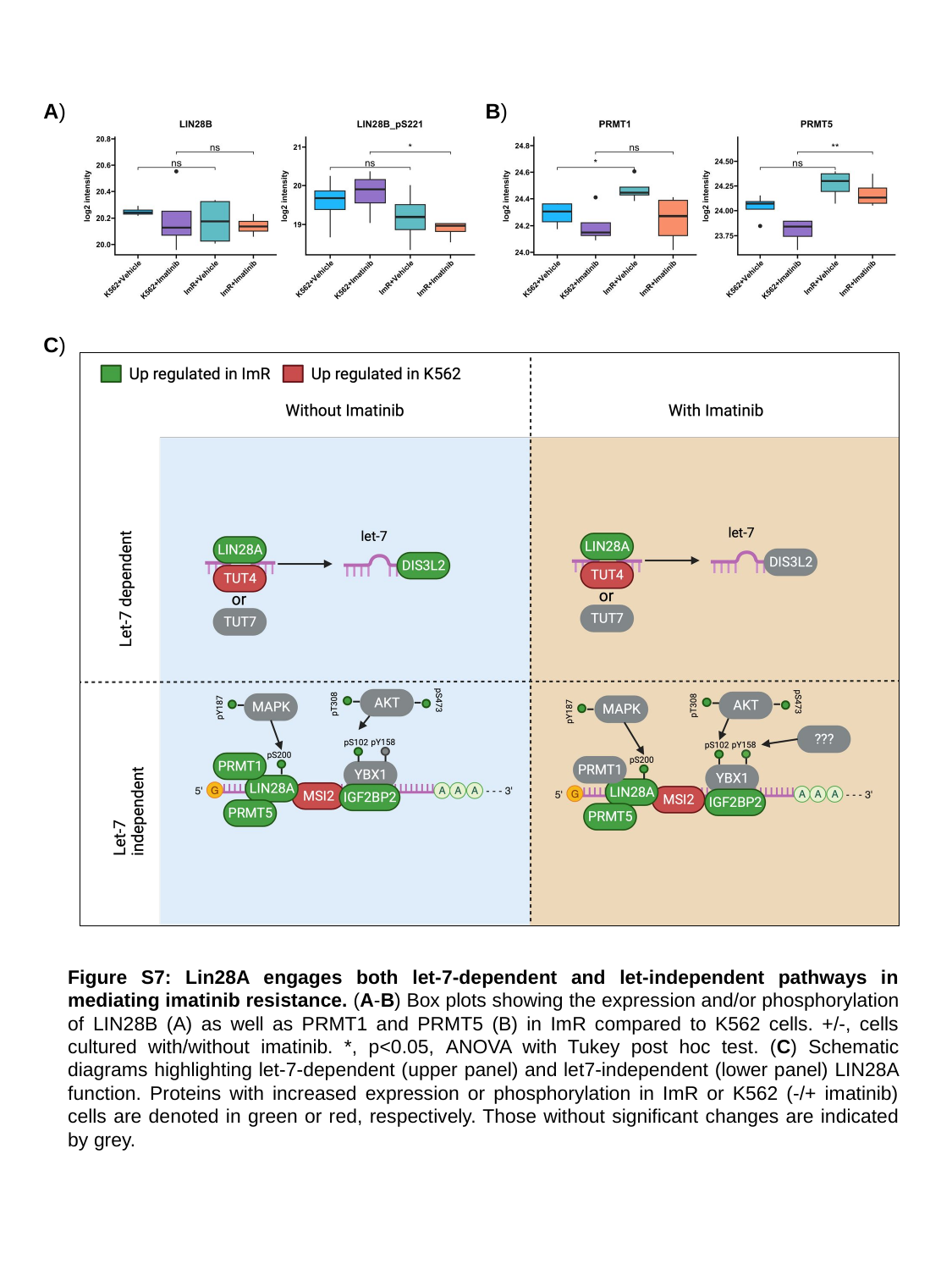

A)
B)
C)
Figure S7: Lin28A engages both let-7-dependent and let-independent pathways in mediating imatinib resistance. (A-B) Box plots showing the expression and/or phosphorylation of LIN28B (A) as well as PRMT1 and PRMT5 (B) in ImR compared to K562 cells. +/-, cells cultured with/without imatinib. *, p<0.05, ANOVA with Tukey post hoc test. (C) Schematic diagrams highlighting let-7-dependent (upper panel) and let7-independent (lower panel) LIN28A function. Proteins with increased expression or phosphorylation in ImR or K562 (-/+ imatinib) cells are denoted in green or red, respectively. Those without significant changes are indicated by grey.

## Slide 8
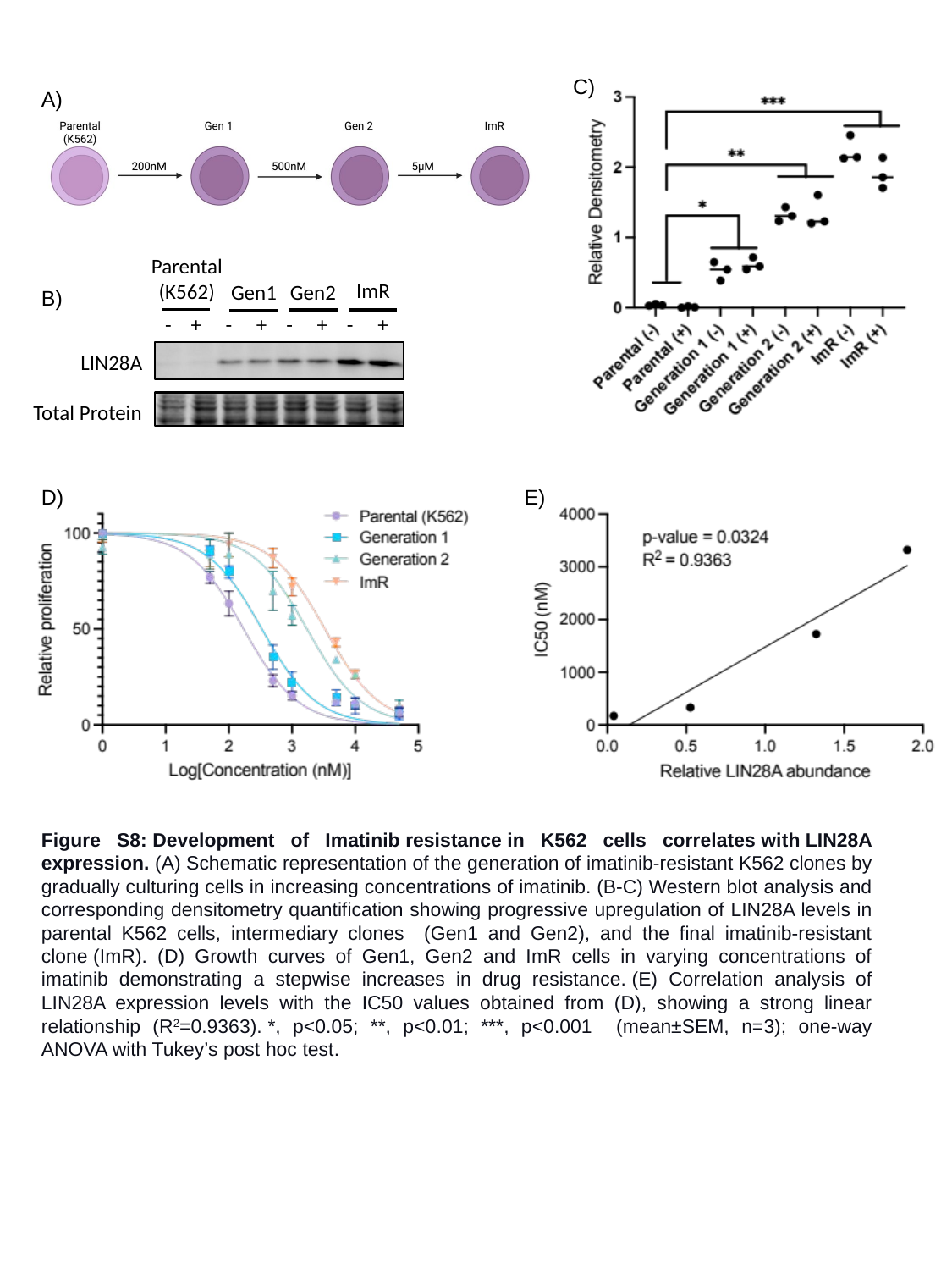

C)
A)
Parental
(K562)
ImR
Gen1
Gen2
B)
- + - + - + - +
LIN28A
Total Protein
E)
D)
Figure S8: Development of Imatinib resistance in K562 cells correlates with LIN28A expression. (A) Schematic representation of the generation of imatinib-resistant K562 clones by gradually culturing cells in increasing concentrations of imatinib. (B-C) Western blot analysis and corresponding densitometry quantification showing progressive upregulation of LIN28A levels in parental K562 cells, intermediary clones (Gen1 and Gen2), and the final imatinib-resistant clone (ImR). (D) Growth curves of Gen1, Gen2 and ImR cells in varying concentrations of imatinib demonstrating a stepwise increases in drug resistance. (E) Correlation analysis of LIN28A expression levels with the IC50 values obtained from (D), showing a strong linear relationship (R2=0.9363). *, p<0.05; **, p<0.01; ***, p<0.001 (mean±SEM, n=3); one-way ANOVA with Tukey’s post hoc test.

## Slide 9
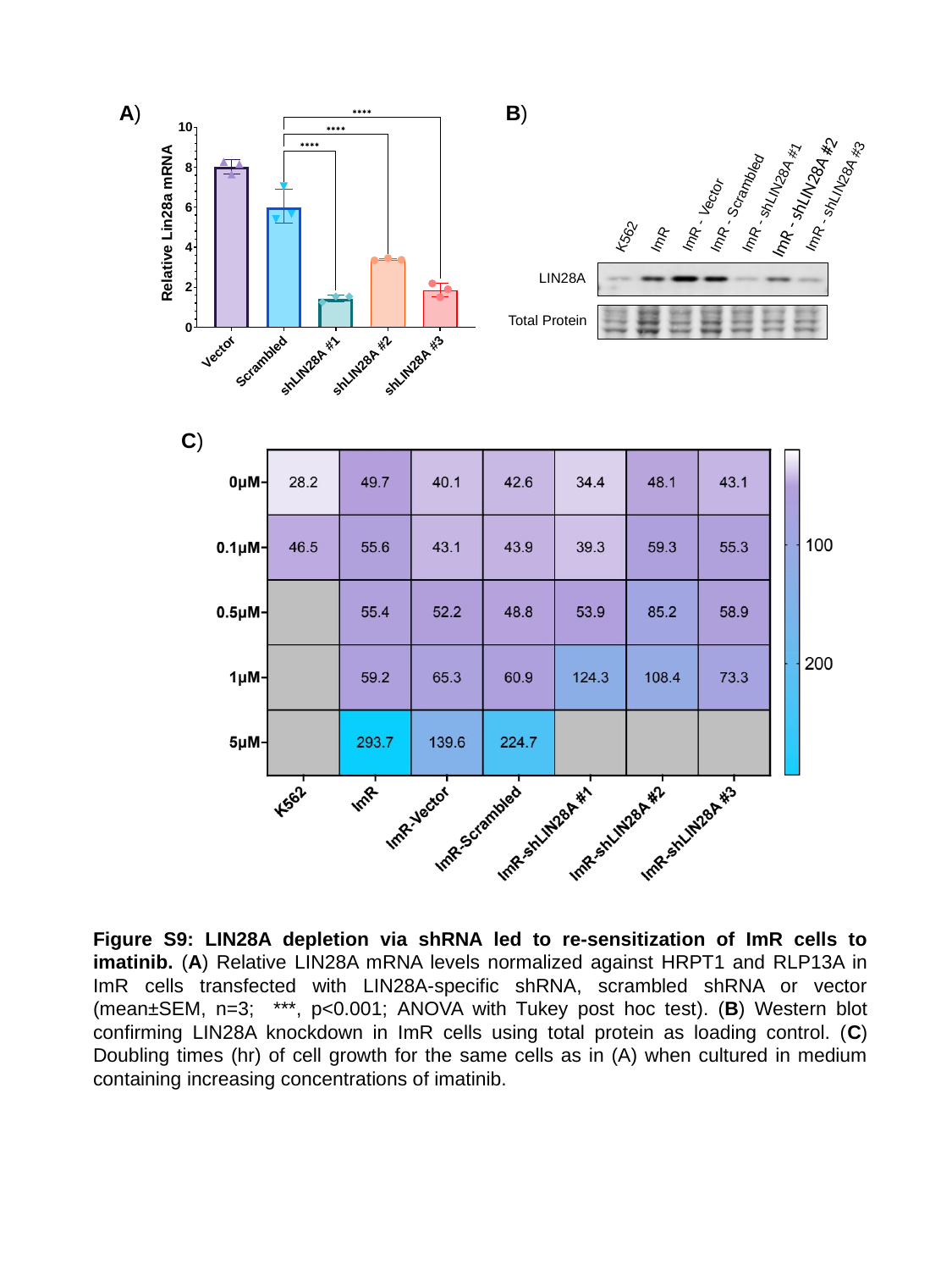

A)
B)
ImR - shLIN28A #2
ImR - shLIN28A #3
ImR - shLIN28A #1
ImR - Scrambled
ImR - Vector
K562
ImR
LIN28A
Total Protein
C)
Figure S9: LIN28A depletion via shRNA led to re-sensitization of ImR cells to imatinib. (A) Relative LIN28A mRNA levels normalized against HRPT1 and RLP13A in ImR cells transfected with LIN28A-specific shRNA, scrambled shRNA or vector (mean±SEM, n=3; ***, p<0.001; ANOVA with Tukey post hoc test). (B) Western blot confirming LIN28A knockdown in ImR cells using total protein as loading control. (C) Doubling times (hr) of cell growth for the same cells as in (A) when cultured in medium containing increasing concentrations of imatinib.

## Slide 10
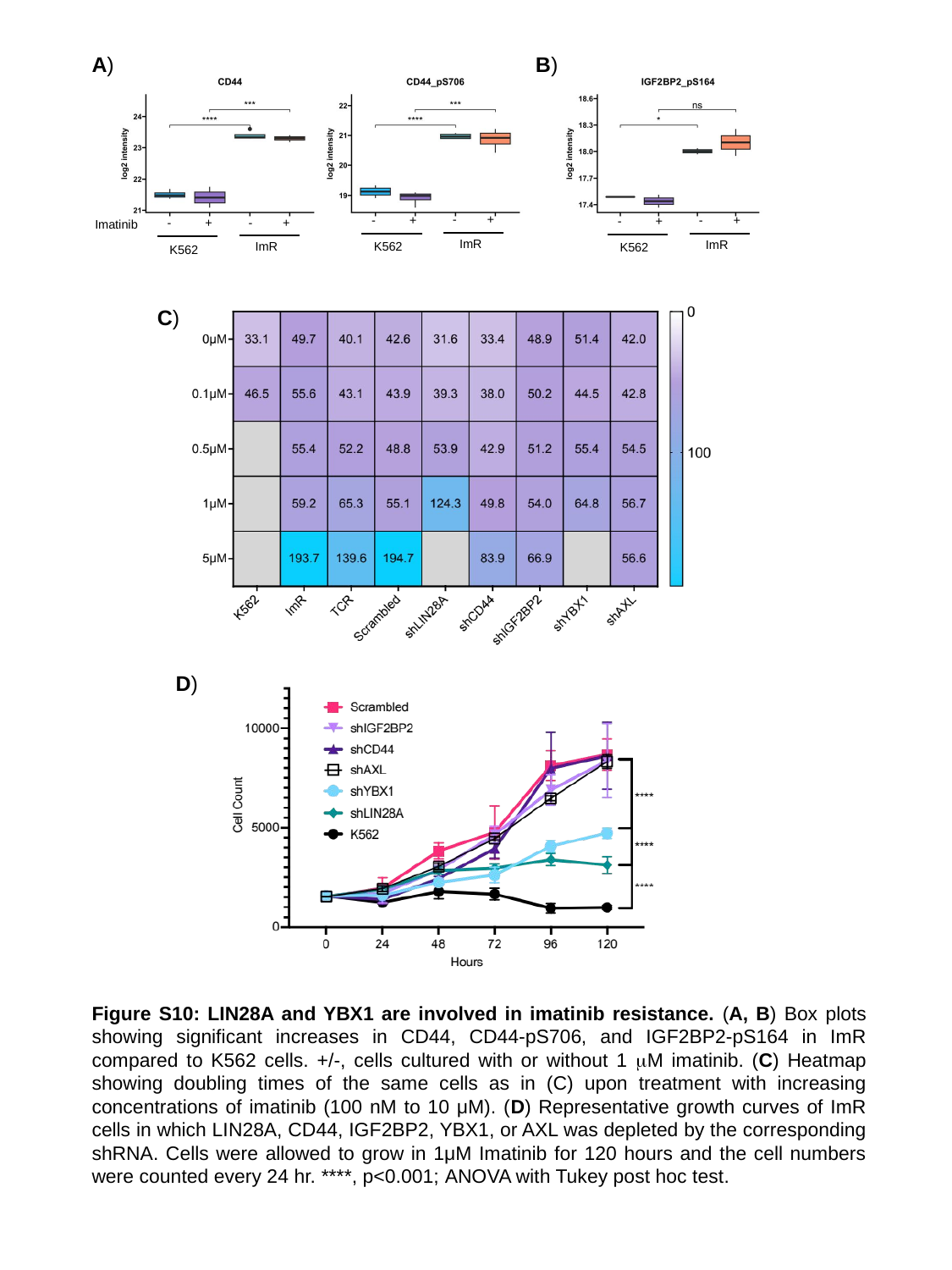

A)
B)
- +
- +
ImR
K562
- +
- +
ImR
K562
- +
- +
ImR
K562
Imatinib
C)
D)
Figure S10: LIN28A and YBX1 are involved in imatinib resistance. (A, B) Box plots showing significant increases in CD44, CD44-pS706, and IGF2BP2-pS164 in ImR compared to K562 cells. +/-, cells cultured with or without 1 mM imatinib. (C) Heatmap showing doubling times of the same cells as in (C) upon treatment with increasing concentrations of imatinib (100 nM to 10 μM). (D) Representative growth curves of ImR cells in which LIN28A, CD44, IGF2BP2, YBX1, or AXL was depleted by the corresponding shRNA. Cells were allowed to grow in 1μM Imatinib for 120 hours and the cell numbers were counted every 24 hr. ****, p<0.001; ANOVA with Tukey post hoc test.

## Slide 11
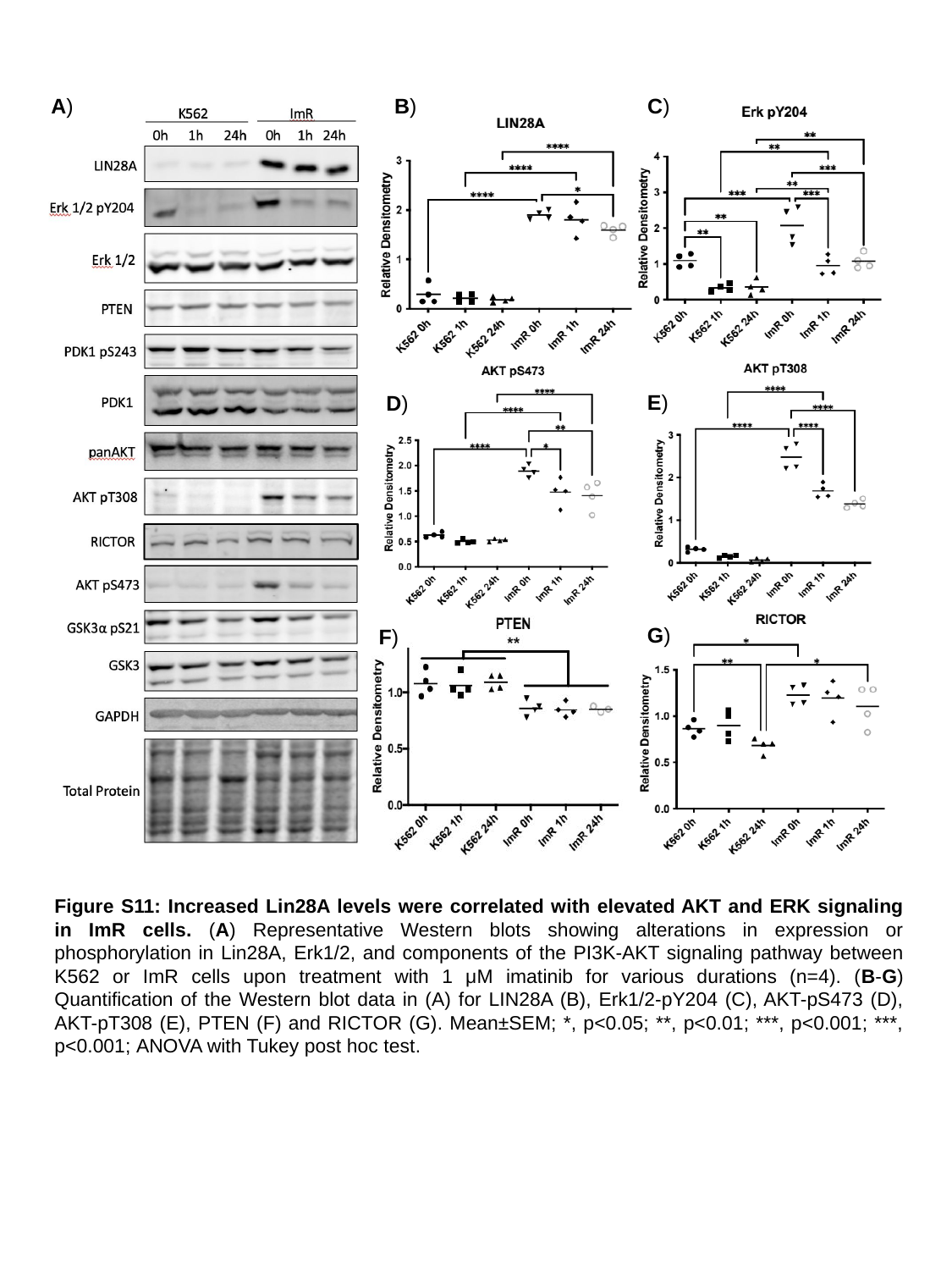

A)
B)
C)
E)
D)
G)
F)
Figure S11: Increased Lin28A levels were correlated with elevated AKT and ERK signaling in ImR cells. (A) Representative Western blots showing alterations in expression or phosphorylation in Lin28A, Erk1/2, and components of the PI3K-AKT signaling pathway between K562 or ImR cells upon treatment with 1 μM imatinib for various durations (n=4). (B-G) Quantification of the Western blot data in (A) for LIN28A (B), Erk1/2-pY204 (C), AKT-pS473 (D), AKT-pT308 (E), PTEN (F) and RICTOR (G). Mean±SEM; *, p<0.05; **, p<0.01; ***, p<0.001; ***, p<0.001; ANOVA with Tukey post hoc test.

## Slide 12
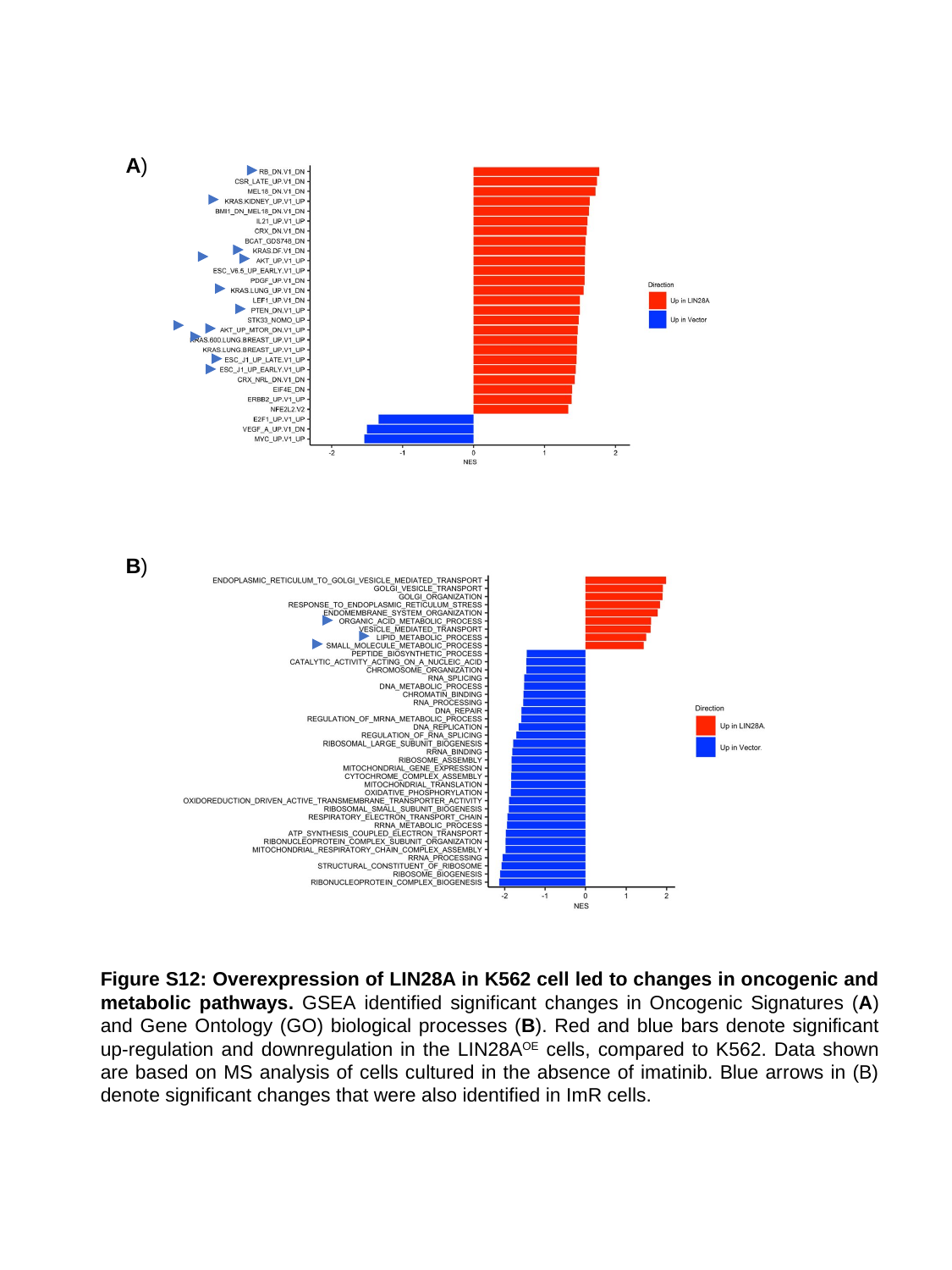

A)
B)
Figure S12: Overexpression of LIN28A in K562 cell led to changes in oncogenic and metabolic pathways. GSEA identified significant changes in Oncogenic Signatures (A) and Gene Ontology (GO) biological processes (B). Red and blue bars denote significant up-regulation and downregulation in the LIN28AOE cells, compared to K562. Data shown are based on MS analysis of cells cultured in the absence of imatinib. Blue arrows in (B) denote significant changes that were also identified in ImR cells.

## Slide 13
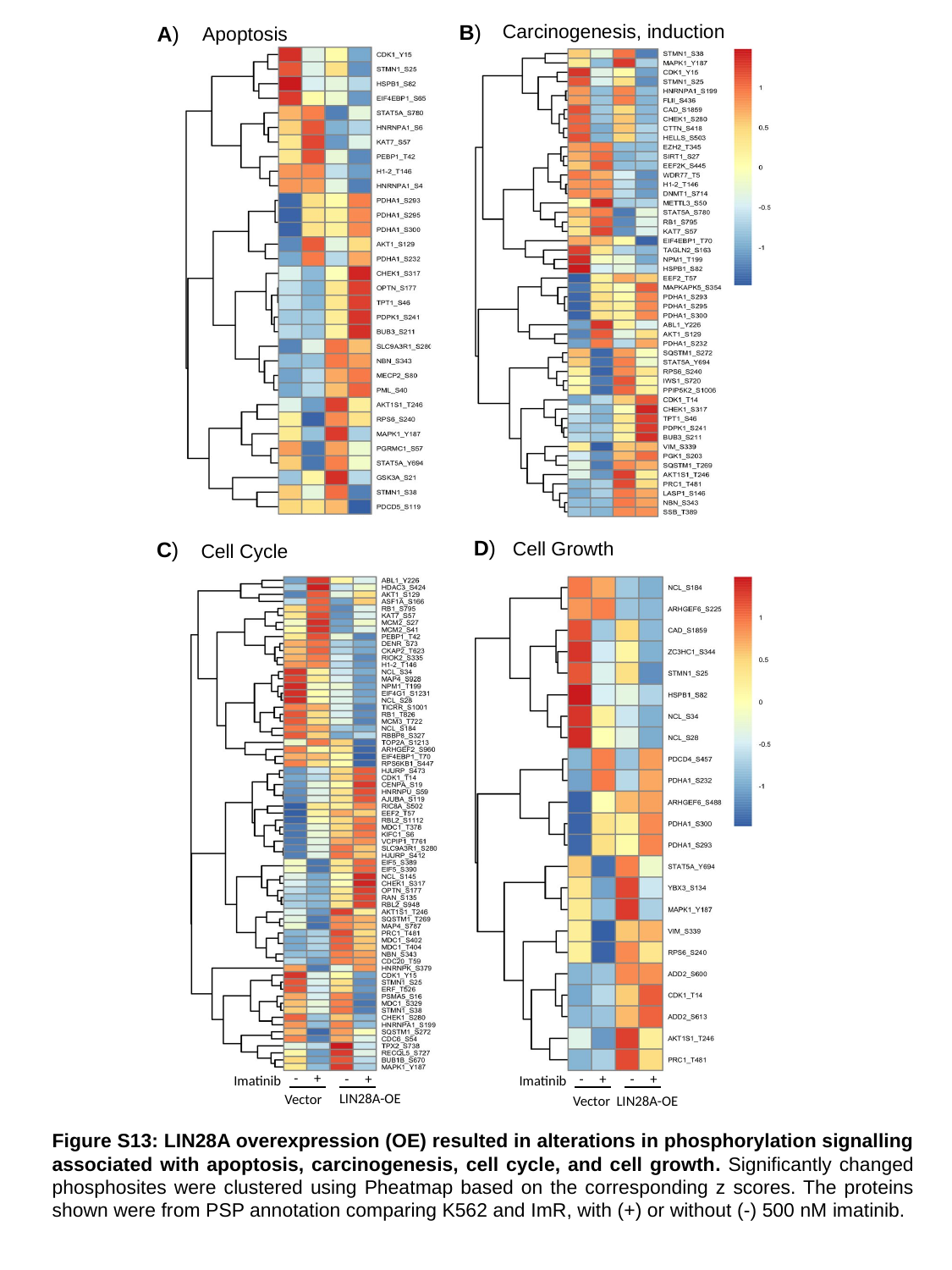

Carcinogenesis, induction
B)
A)
Apoptosis
D)
C)
Cell Growth
Cell Cycle
- +
- +
- +
- +
Imatinib
Imatinib
LIN28A-OE
Vector
Vector
LIN28A-OE
Figure S13: LIN28A overexpression (OE) resulted in alterations in phosphorylation signalling associated with apoptosis, carcinogenesis, cell cycle, and cell growth. Significantly changed phosphosites were clustered using Pheatmap based on the corresponding z scores. The proteins shown were from PSP annotation comparing K562 and ImR, with (+) or without (-) 500 nM imatinib.

## Slide 14
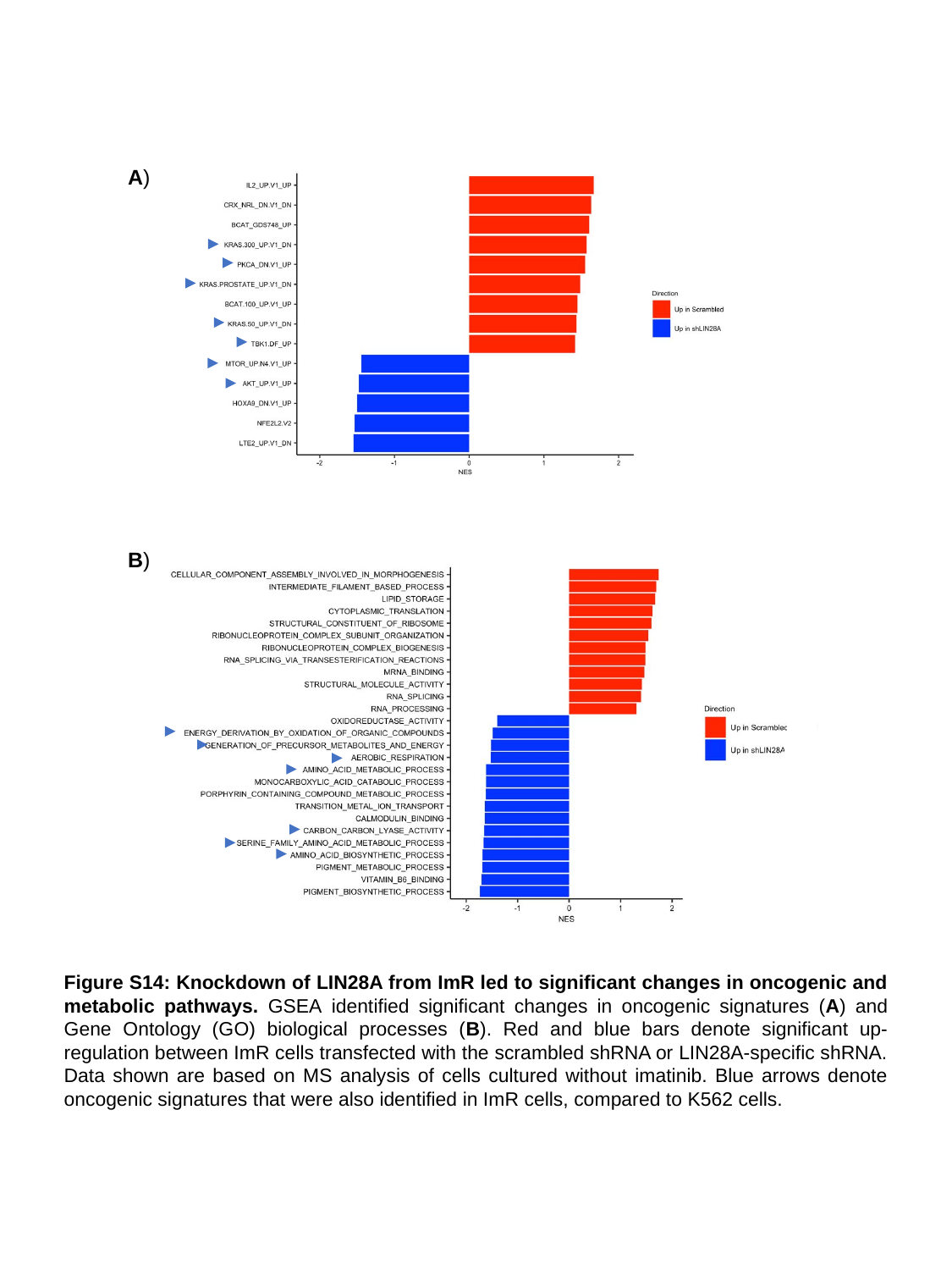

A)
B)
Figure S14: Knockdown of LIN28A from ImR led to significant changes in oncogenic and metabolic pathways. GSEA identified significant changes in oncogenic signatures (A) and Gene Ontology (GO) biological processes (B). Red and blue bars denote significant up-regulation between ImR cells transfected with the scrambled shRNA or LIN28A-specific shRNA. Data shown are based on MS analysis of cells cultured without imatinib. Blue arrows denote oncogenic signatures that were also identified in ImR cells, compared to K562 cells.

## Slide 15
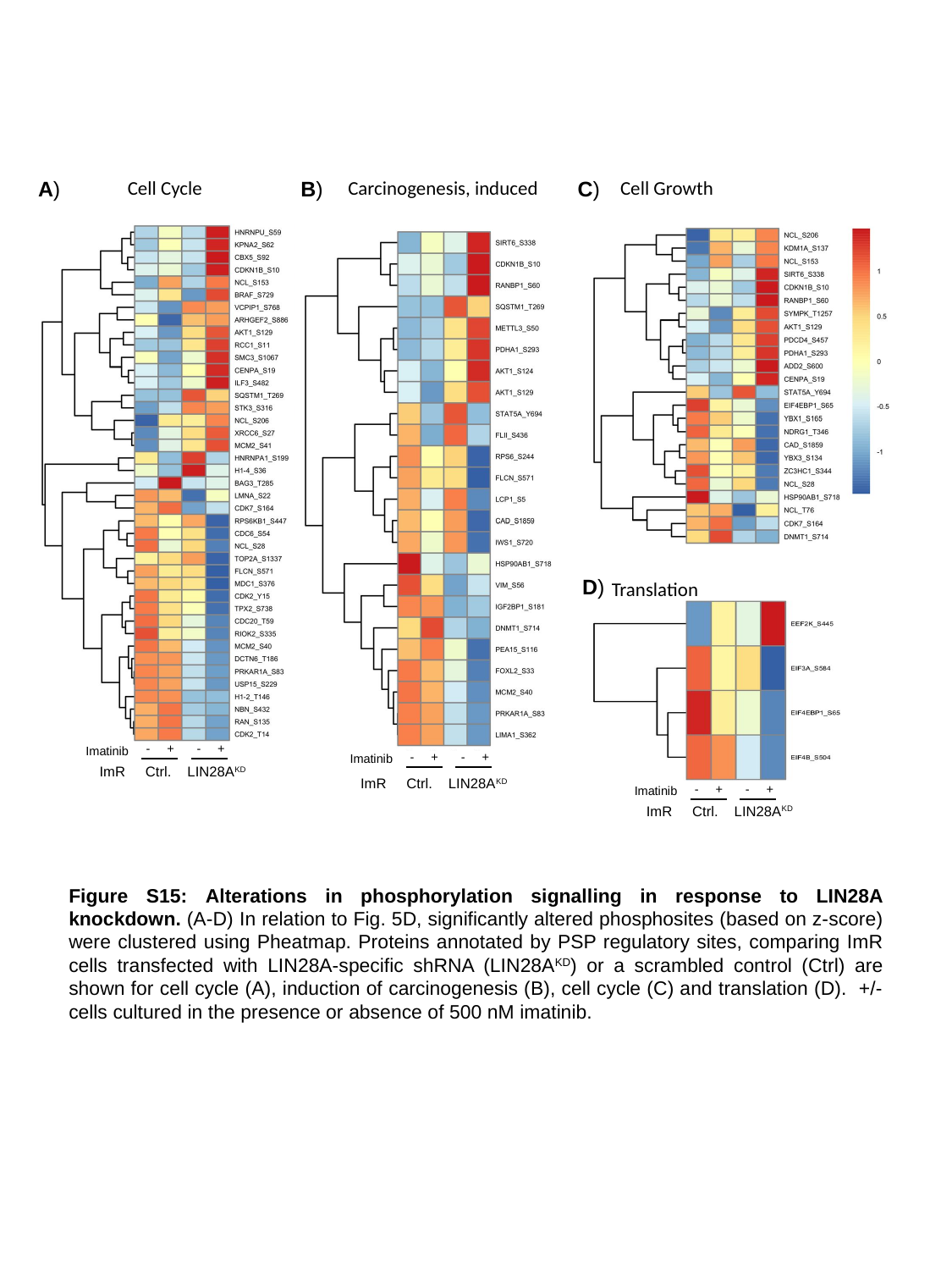

Cell Cycle
A)
B)
C)
Cell Growth
Carcinogenesis, induced
D)
Translation
- +
- +
Imatinib
ImR Ctrl. LIN28AKD
- +
- +
Imatinib
ImR Ctrl. LIN28AKD
- +
- +
Imatinib
ImR Ctrl. LIN28AKD
Figure S15: Alterations in phosphorylation signalling in response to LIN28A knockdown. (A-D) In relation to Fig. 5D, significantly altered phosphosites (based on z-score) were clustered using Pheatmap. Proteins annotated by PSP regulatory sites, comparing ImR cells transfected with LIN28A-specific shRNA (LIN28AKD) or a scrambled control (Ctrl) are shown for cell cycle (A), induction of carcinogenesis (B), cell cycle (C) and translation (D). +/- cells cultured in the presence or absence of 500 nM imatinib.

## Slide 16
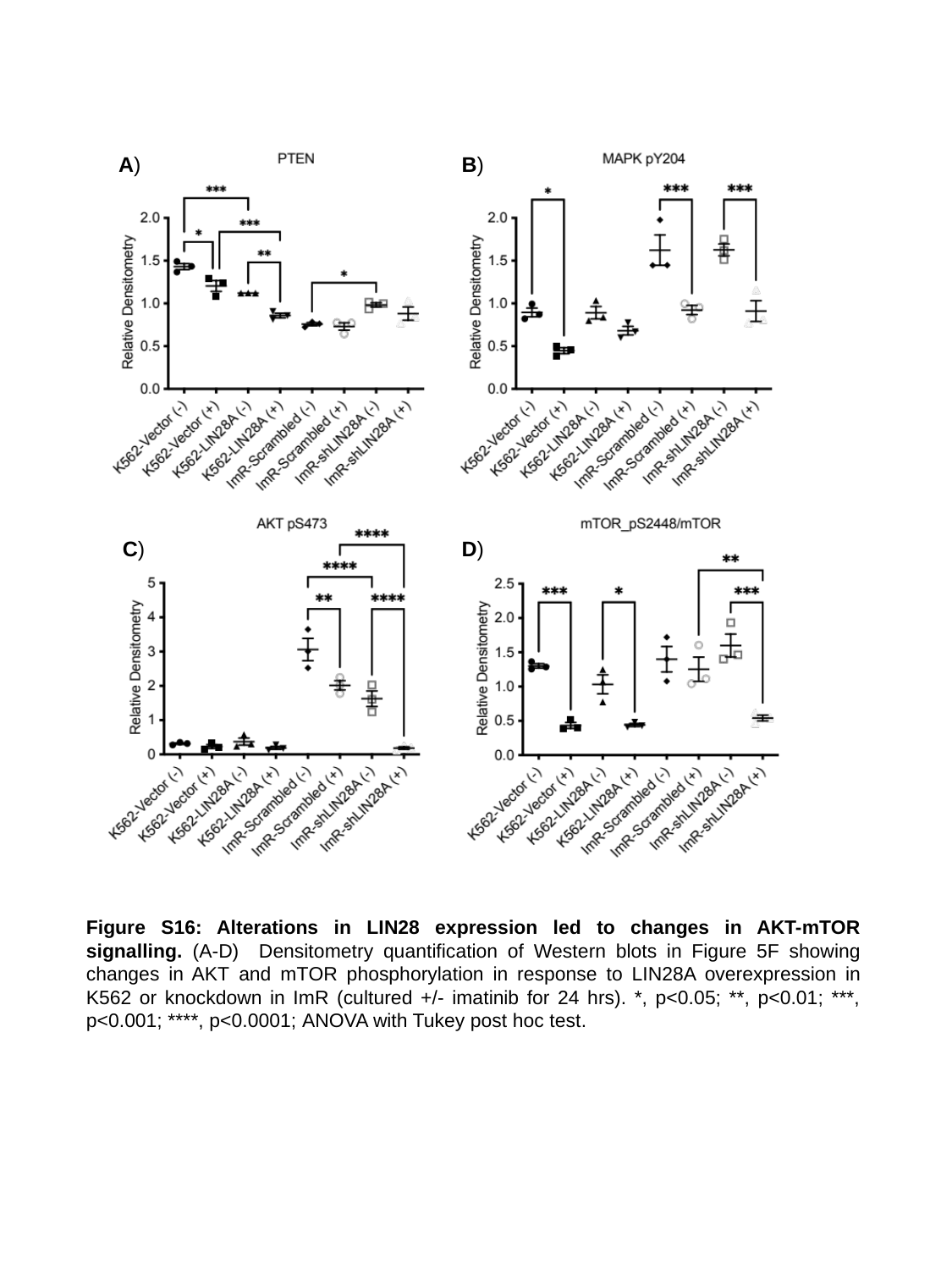

B)
A)
C)
D)
Figure S16: Alterations in LIN28 expression led to changes in AKT-mTOR signalling. (A-D) Densitometry quantification of Western blots in Figure 5F showing changes in AKT and mTOR phosphorylation in response to LIN28A overexpression in K562 or knockdown in ImR (cultured +/- imatinib for 24 hrs). *, p<0.05; **, p<0.01; ***, p<0.001; ****, p<0.0001; ANOVA with Tukey post hoc test.

## Slide 17
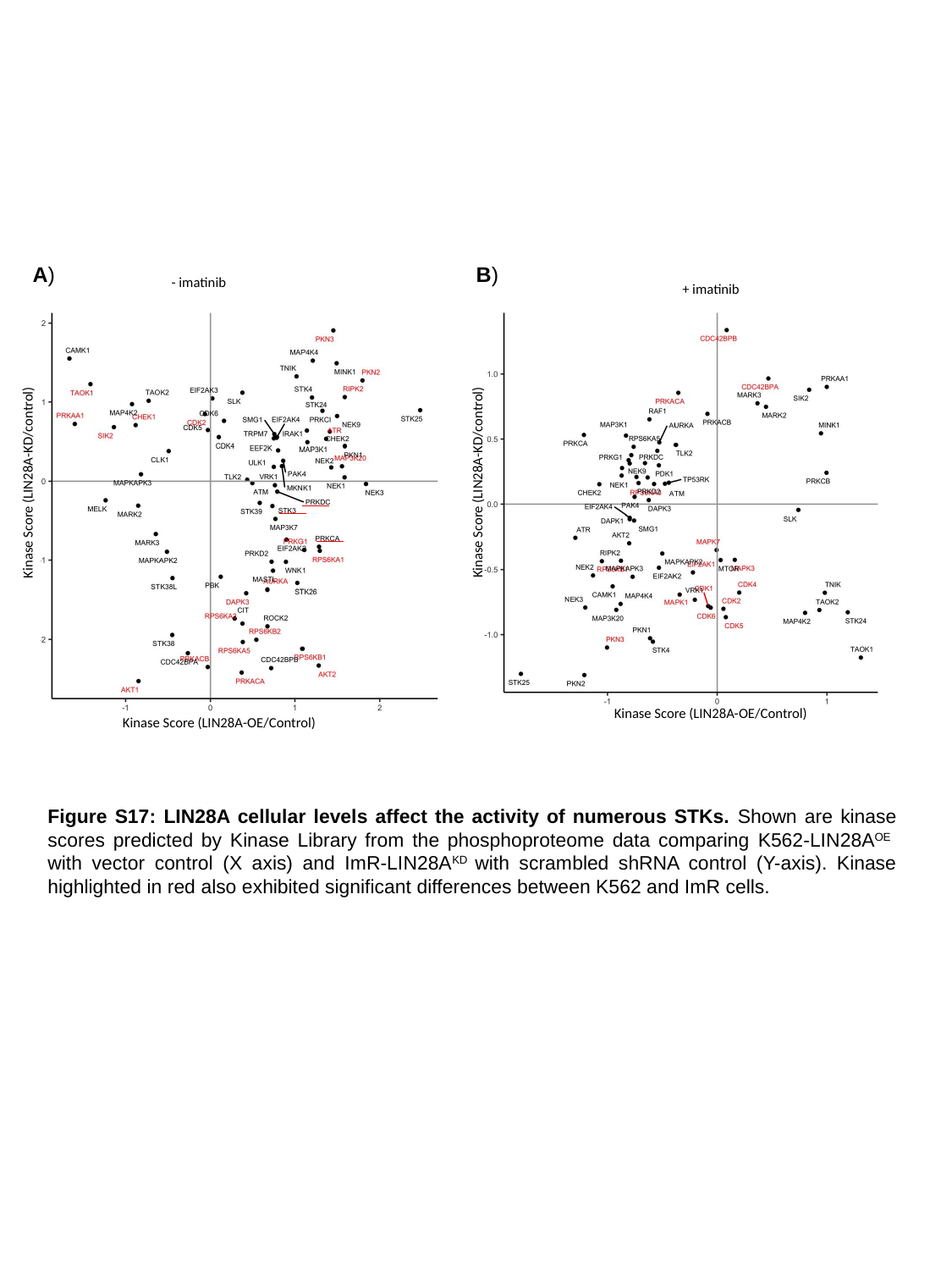

A)
B)
- imatinib
+ imatinib
Kinase Score (LIN28A-KD/control)
Kinase Score (LIN28A-KD/control)
Kinase Score (LIN28A-OE/Control)
Kinase Score (LIN28A-OE/Control)
Figure S17: LIN28A cellular levels affect the activity of numerous STKs. Shown are kinase scores predicted by Kinase Library from the phosphoproteome data comparing K562-LIN28AOE with vector control (X axis) and ImR-LIN28AKD with scrambled shRNA control (Y-axis). Kinase highlighted in red also exhibited significant differences between K562 and ImR cells.

## Slide 18
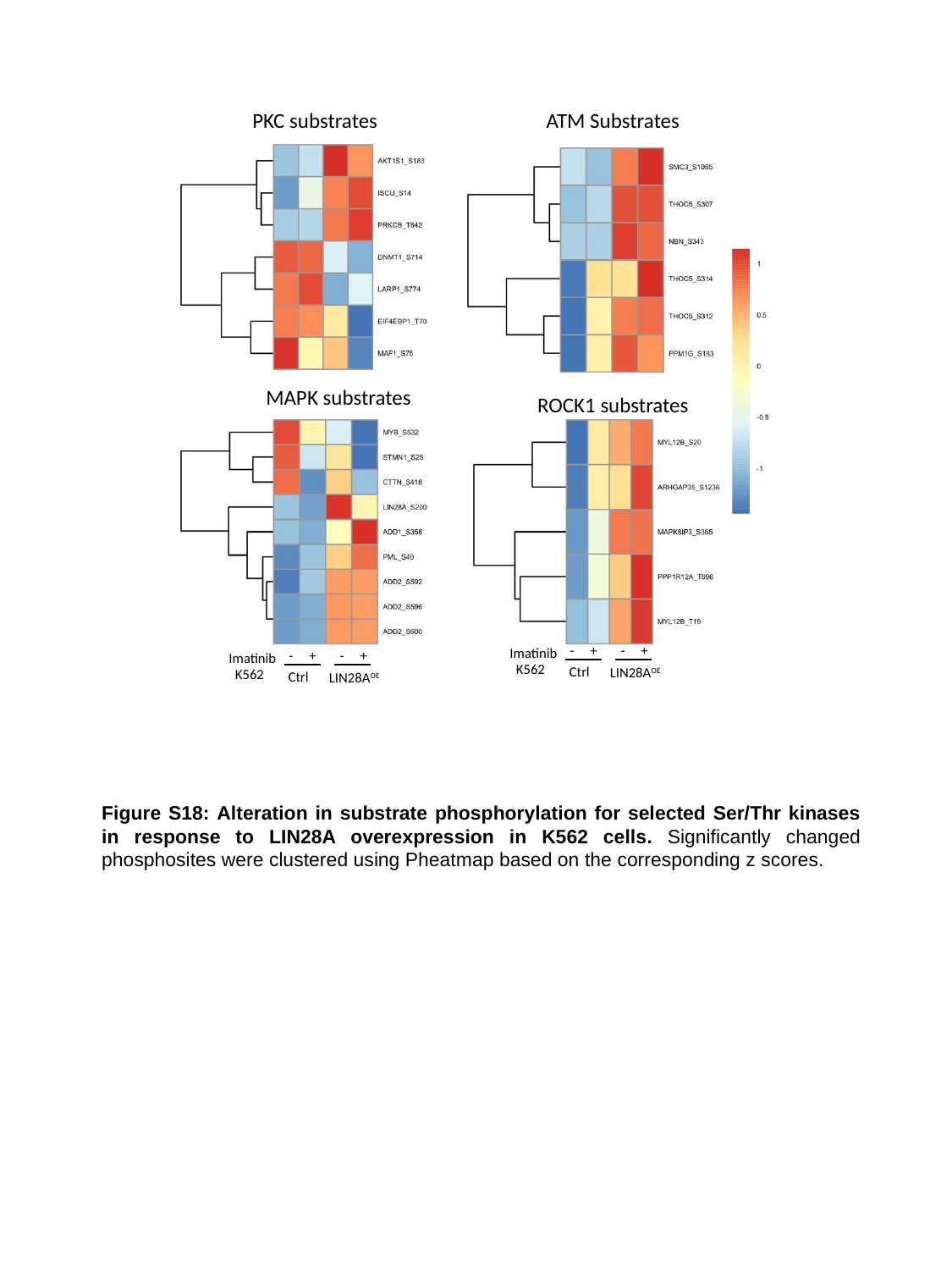

PKC substrates
ATM Substrates
MAPK substrates
ROCK1 substrates
- +
- +
Imatinib
- +
- +
Imatinib
K562
Ctrl
LIN28AOE
K562
Ctrl
LIN28AOE
Figure S18: Alteration in substrate phosphorylation for selected Ser/Thr kinases in response to LIN28A overexpression in K562 cells. Significantly changed phosphosites were clustered using Pheatmap based on the corresponding z scores.

## Slide 19
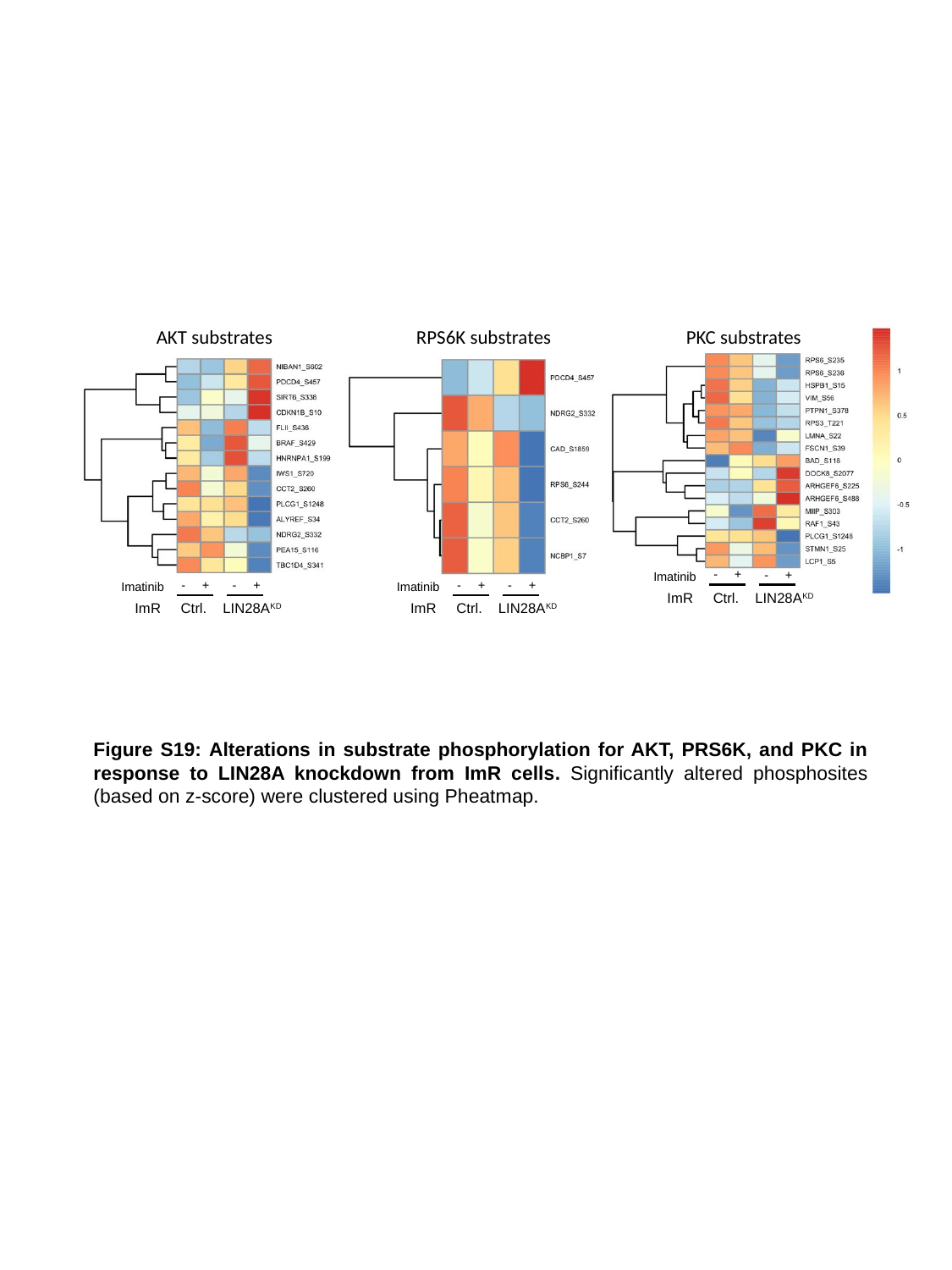

AKT substrates
RPS6K substrates
PKC substrates
- +
- +
Imatinib
ImR Ctrl. LIN28AKD
- +
- +
Imatinib
ImR Ctrl. LIN28AKD
- +
- +
Imatinib
ImR Ctrl. LIN28AKD
Figure S19: Alterations in substrate phosphorylation for AKT, PRS6K, and PKC in response to LIN28A knockdown from ImR cells. Significantly altered phosphosites (based on z-score) were clustered using Pheatmap.

## Slide 20
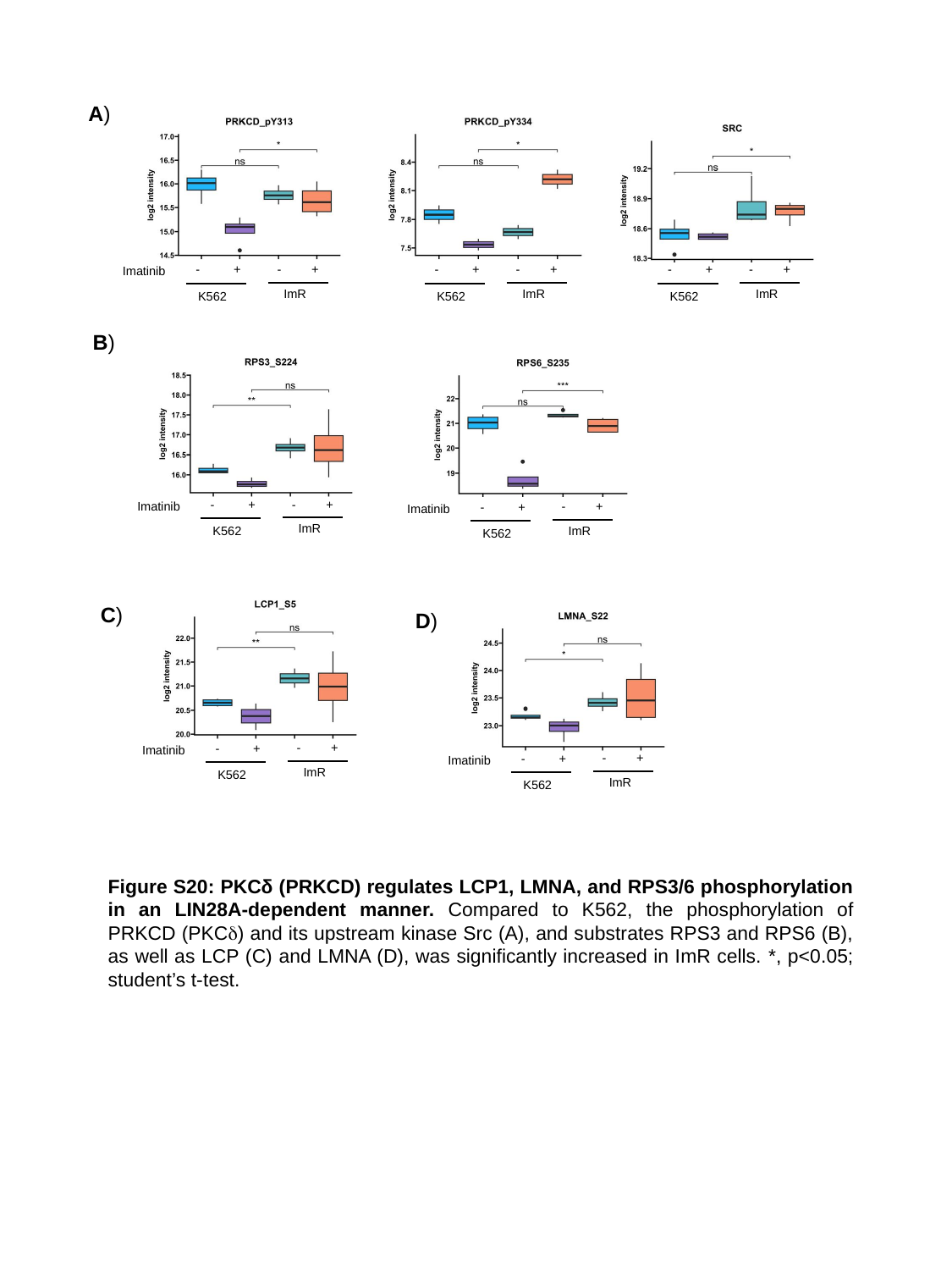

A)
- +
- +
ImR
K562
- +
- +
ImR
K562
Imatinib
- +
- +
ImR
K562
B)
- +
- +
ImR
K562
Imatinib
- +
- +
ImR
K562
Imatinib
C)
- +
- +
ImR
K562
Imatinib
D)
- +
- +
ImR
K562
Imatinib
Figure S20: PKCδ (PRKCD) regulates LCP1, LMNA, and RPS3/6 phosphorylation in an LIN28A-dependent manner. Compared to K562, the phosphorylation of PRKCD (PKCd) and its upstream kinase Src (A), and substrates RPS3 and RPS6 (B), as well as LCP (C) and LMNA (D), was significantly increased in ImR cells. *, p<0.05; student’s t-test.

## Slide 21
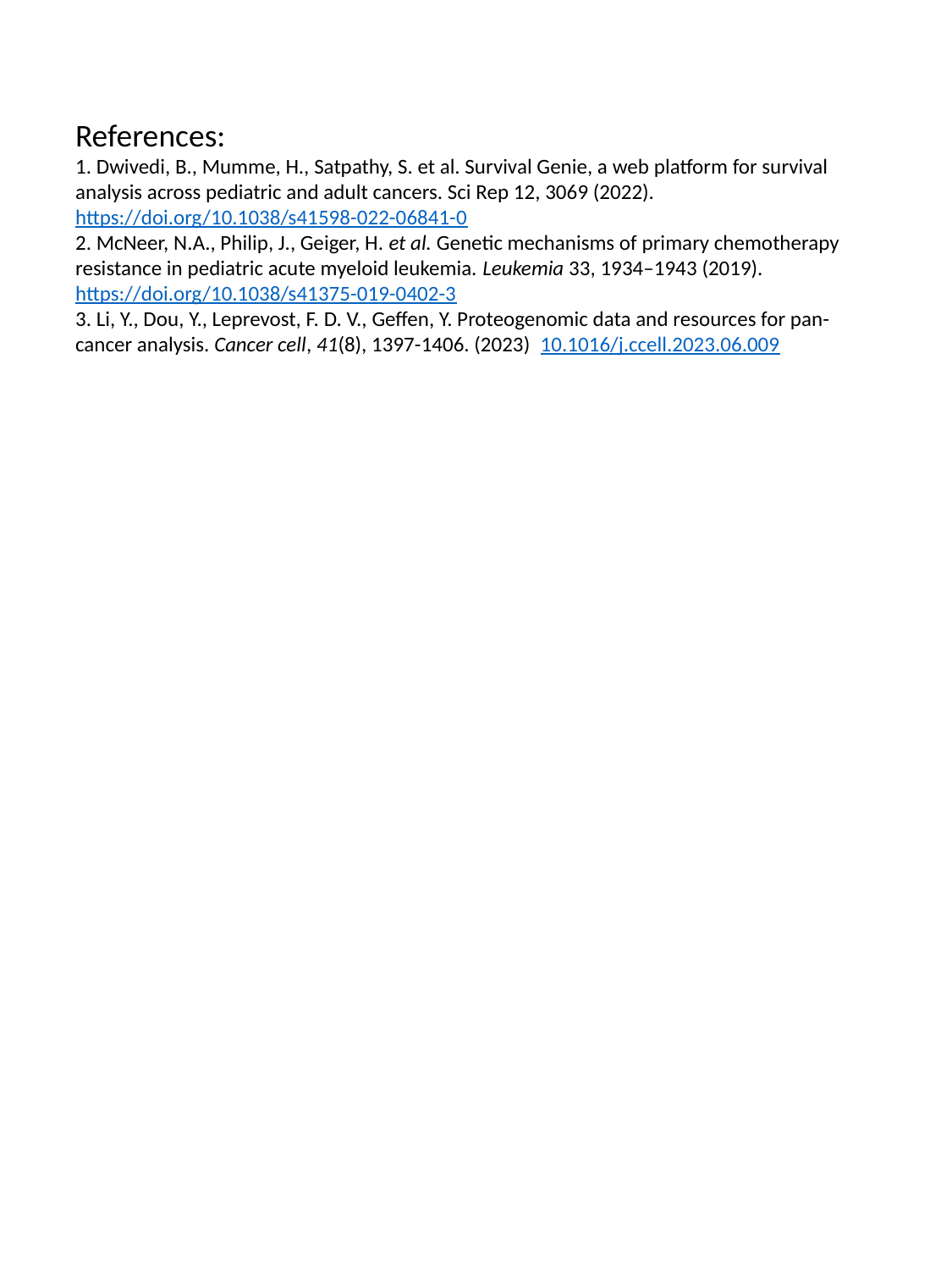

References:
1. Dwivedi, B., Mumme, H., Satpathy, S. et al. Survival Genie, a web platform for survival analysis across pediatric and adult cancers. Sci Rep 12, 3069 (2022). https://doi.org/10.1038/s41598-022-06841-0
2. McNeer, N.A., Philip, J., Geiger, H. et al. Genetic mechanisms of primary chemotherapy resistance in pediatric acute myeloid leukemia. Leukemia 33, 1934–1943 (2019). https://doi.org/10.1038/s41375-019-0402-3
3. Li, Y., Dou, Y., Leprevost, F. D. V., Geffen, Y. Proteogenomic data and resources for pan-cancer analysis. Cancer cell, 41(8), 1397-1406. (2023)  10.1016/j.ccell.2023.06.009
